# ^68^Ga-PSMA-11 NDA Approval: A Novel and Successful Academic Partnership

**DOI:** 10.2967/jnumed.120.260455

**Published:** 2021-02

**Authors:** Giuseppe Carlucci, Robin Ippisch, Roger Slavik, Ashley Mishoe, Joseph Blecha, Shaojun Zhu

**Affiliations:** 1 Ahmanson Translational Theranostics Division, Department of Molecular and Medical Pharmacology, David Geffen School of Medicine, University of California, Los Angeles, Los Angeles, California; and; 2 Department of Radiology and Biomedical Imaging, University of California, San Francisco, San Francisco, California

**Keywords:** oncology: GU, PET/CT, radiochemistry, radiopharmaceuticals, ^68^Ga-PSMA-11, approval, FDA, new drug application

## Abstract

The University of California Los Angeles (UCLA) and University of California San Francisco (UCSF) codeveloped ^68^Ga-PSMA-11 by conducting a bicentric pivotal phase 3 clinical trial for PET imaging for prostate cancer. On December 1, 2020, 2 separate new drug applications (NDAs) submitted by each institution (NDA 212642 for UCLA and NDA 212643 for UCSF) were approved by the Food and Drug Administration as the first drug for PET imaging of prostate-specific membrane antigen (PSMA)–positive lesions in men with prostate cancer. This article briefly describes the background, clinical development, regulatory approach, and regulatory process for NDA filing and approval. In the second part of this article, key chemistry, manufacturing, and controls (CMC) information is provided to facilitate abbreviated new drug application (ANDA) submission.

**NOTEWORTHY**

- The unique NDA approach (2 separate NDAs sharing the same clinical and nonclinical information) paved the way for collaborative PET drug development by academic institutions.
- Both NDAs waived market exclusivity, so that the PET imaging providers can submit an ANDA immediately, using either NDA drug product as a reference drug.
- Academic institutions are indispensable for regulatory approval of PET drugs, whereas regular drug development and commercialization depends nearly exclusively on industry sponsorship.

PET imaging with ^68^Ga-PSMA-11 has been adopted in many countries for initial staging and restaging and for localizing biochemical recurrence of prostate cancers. In the United States, it was under investigational use until December 1, 2020, when University of California San Francisco (UCSF, New Drug Application [NDA] 212643) and University of California Los Angeles (UCLA, NDA 212642) received Food and Drug Administration (FDA) approval for both NDAs.

Led by the sponsor-investigators from both institutions (Drs. Thomas Hope–UCSF and Johannes Czernin–UCLA), the NDA process was based on a highly collaborative effort by a multidisciplinary and interinstitutional team (nuclear medicine, radiochemistry, urology, radiation oncology, radiology, and regulatory affairs) supported by the Society of Nuclear Medicine and Molecular Imaging Clinical Trials Network.

This article describes the background and regulatory pathway for these NDAs, the key drug development milestones and regulatory interactions, and the implications to the PET community. It also provides the key Chemistry, Manufacturing and Controls (CMC) information for ^68^Ga-PSMA-11 to facilitate abbreviated NDA (ANDA) submission.

## PART I: ^68^GA-PSMA-11 DEVELOPMENT AND REGULATORY APPROVAL PROCESS

### ^68^Ga-PSMA-11 Background and Regulatory Pathway

Prostate-specific membrane antigen (PSMA)–targeted PET imaging has received increased attention (1) and is clinically used in many countries (2,3). Use of ^68^Ga-labeled PSMA ligands has been embraced enthusiastically worldwide (4). ^68^Ga-PSMA-11 was developed by investigators from the German Cancer Research Center and Heidelberg University and was shared with the scientific community free of patent.

In the United States, some academic institutions perform PSMA-targeted PET imaging under effective Investigational New Drug (IND) applications with FDA’s approval to recover the manufacturing cost of the probe from patients (Title 21 of the Code of Federal Regulation section 312.8). However, this approach posed a significant financial toxicity to the patients because ^68^Ga-PSMA-11 was investigational only and was reimbursed by neither Medicare nor private health insurance companies. Given the unmet clinical need for accurately staging and restaging prostate cancer as well as detecting sites of biochemical recurrence and lack of industry support, we initiated a pivotal phase 3 clinical trial for ^68^Ga-PSMA-11 to study its efficacy and safety in prostate cancer detection.

At the beginning of 2018, the UCSF and UCLA teams consulted with the FDA Division of Medical Imaging and Radiation Medicine to develop a unique regulatory approach for the NDA submission. UCSF and UCLA would submit 2 separate NDAs around the same time for the same PET drug, waiving market exclusivity. Both NDAs would share the same nonclinical and clinical information, similar labeling information, but with site-specific CMC modules. Both submissions would be 505(b)(2) NDAs, referencing published literature for nonclinical and clinical pharmacology and clinical dosimetry. Clinical information was analyzed jointly by both centers. In addition, a metaanalysis of published literature of relevant clinical trials would serve as confirmatory evidence, corroborating the clinical data derived from the bicentric, prospective pivotal trial conducted by UCSF and UCLA.

### Key Drug Development Milestones and Regulatory Interactions with the FDA

In late 2016, pivotal phase 3 clinical trial protocols were submitted to IND application 127621 (UCSF) and 130649 (UCLA). Uniform clinical protocols were designed and followed to collect safety and efficacy data. Even though the ^68^Ga-PSMA-11 was supplied locally by UCLA and UCSF radiochemistry facilities, they were produced and controlled in a similar way, resulting in highly compatible final products. At the end of 2017, UCSF and UCLA teams assessed the clinical data and determined that they were adequate for NDA filing. A pre-NDA meeting request and briefing package were filed with the FDA in the spring of 2018. In August 2018, a joint pre-NDA meeting was held on the FDA Silver Spring campus. At the meeting, the data assembled in the briefing package were deemed substantive for NDA review. Furthermore, FDA offered positive feedback and constructive recommendations for the NDA submission plan.

After the pre-NDA meeting, the UCSF/UCLA team submitted Prescription Drug User Fee Act (PDUFA) user application fee waiver requests based on Barrier-to-Innovation provision for both NDAs (PDUFA section 736(d)(1)(B)). In late 2018, both fee waiver requests were granted by the FDA. Without a waiver, PDUFA application fees for the NDA with clinical data would be around $2.6 million each for that fiscal year.

In the following months, a multidisciplinary team consisting of regulatory, nonclinical, clinical, statistical, and CMC experts performed clinical data analyses, registration batch and method/process validations. On September 6, 2019, NDA 212643 (UCSF) and NDA 212642 (UCLA) were submitted to the FDA. Both NDAs received FDA’s Day-74 letters, in which FDA determined that both NDAs were sufficiently complete in content and format to permit a substantive review of the application. The initial PDUFA goal date was September 6, 2020. The goal date was later extended to December 6, 2020, due to major amendments during the NDA review.

During the NDA review, multiple iterations of clinical, statistical, CMC, and labeling information requests were issued by the FDA. Both UCSF and UCLA nuclear medicine clinics and PET manufacturing facilities were inspected by the FDA in 2020.

On December 1, 2020, the FDA approved both NDAs for PET imaging of PSMA-positive lesions in men with prostate cancer:

- with suspected metastasis who are candidates for initial definitive therapy.
- with suspected recurrence based on elevated serum prostate-specific antigen (PSA) level.

The approved package inserts can be accessed via the FDA website (https://www.accessdata.fda.gov/drugsatfda_docs/label/2020/212642s000lbl.pdf; https://www.accessdata.fda.gov/drugsatfda_docs/label/2020/212643s000lbl.pdf)

### Implications to the PET Community

In this joint effort, the UCSF/UCLA team obtained the approval of the first drug for PET imaging of PSMA-positive lesions in men with prostate cancer. These NDAs are based on academic initiatives for a radiopharmaceutical without patent protection.

Since market exclusivities were waived for both NDAs, the PET imaging providers can submit ANDAs immediately, using either drug product as reference drug. PET drug ANDAs are exempt from Generic Drug User Fee Act (GDUFA) user fees and are subject to a 10-mo GDUFA goal date. A detailed outline of the ANDA contents can be found in the FDA’s “Guidance for Industry: ANDA Submissions—Content and Format” (June 2019, https://www.fda.gov/media/128127/download), and FDA Guidance “PET Drug Applications—Content and Format for NDAs and ANDAs” (August 2011, https://www.fda.gov/media/72271/download).

In October 2020, the FDA published a guidance document titled “Referencing Approved Drug Products in ANDA Submissions” (https://www.fda.gov/media/102360/download), which outlines how to choose reference drug and describes the basis for ANDA submissions.

An ANDA submission will most likely only include Module 1 (administrative and labeling information), Module 2 (Common Technical Document [CTD] summary), and Module 3 (CMC information; key information is provided in Part II of this article). Module 1, section 1.12.11, contains the reference drug information, where section 1.12.12 requires information demonstrating that the generic product is the same as the reference drug. PET providers can support the ANDA by demonstrating that the active ingredients, route of administration, dosage form, strength, and conditions of use are the same as those of the reference drug.

In March 2020, the FDA issued eCTD guidance document revision 7, titled “Providing Regulatory Submissions in Electronic Format—Certain Human Pharmaceutical Product Applications and Related Submissions Using the eCTD Specifications” (https://www.fda.gov/media/135373/download). Section III D of this guidance document outlined a possible long-term eCTD waiver pathway for certain PET NDA/ANDA submissions from qualified PET drug facilities. On the basis of our experience, it is feasible to submit long-term waivers and propose alternative submission format. The alternative submission format could be files in PDF format, with content organized by folders and subfolders mirroring the eCTD structure. The final ANDA application can be submitted via the Center for Drug Evaluation and Research Nextgen Portal (http://edm.fda.gov).

## PART II: KEY CMC INFORMATION FOR ^68^GA-PSMA-11

UCSF and UCLA NDAs share the similar body of CMC data, with some minor differences. UCSF uses the Eckert and Ziegler (E&Z) GalliaPharm ^68^Ge/^68^Ga Generator or cyclotron-produced ^68^Ga, whereas UCLA uses only the E&Z GalliaPharm Generator for ^68^Ga. The key CMC information for both NDAs is provided below.

### Drug Substance

#### 3.2.S.1 General Information

General properties are given, with a short description to highlight nomenclature, physical structure characteristics, and external radiation.

#### 3.2.S.2 Manufacture

PSMA-11 precursor is supplied and manufactured by ABX Advanced Biochemical Compounds Biochemische Forschungsreagenzien GmbH.

#### 3.2.S.3 Characterization

The chemical purity and identity of the incoming PSMA-11 precursor are tested on receipt. PSMA-11 precursor is released for clinical use based on Certificates of Analysis (CoA) provided by the supplier and passing results from the in-house acceptance testing.

#### 3.2.S.4 Control of Drug Substance

The specifications for the PSMA-11 precursor are summarized in Table 1. The details of the precursor specification are detailed within the Drug Master File (DMF) 033682 from ABX.

**TABLE 1:** PSMA-11 Precursor Specifications

Criteria	Acceptance
Specific filling	
Amount of filling	1 mg ± 10%
Identity (HPLC)	Reference retention time ± 0.5 min
Impurities (HPLC)	Any unspecified impurity ≤ 2.0%
	Total impurities ≤ 6.0%
Bioburden/vial	TAMC ≤ 100 cfu
	TYMC ≤ 10 cfu
BET (LAL)	≤ 10 IU/vial
Bulk precursor batch	
Appearance	White to off-white solid
Monoisotopic mass (net peptide)	946.1 ± 1 m.u.
^1^H-NMR	Conforms to structure
^13^C-NMR	Conforms to structure
Impurities (HPLC)	Any unspecified impurity ≤ 2.0%
	Total impurities ≤ 3.0%
Residual solvents (GC)	Acetonitrile ≤ 5,000 ppm
	Tertbutylmethylether ≤ 5,000 ppm
Water (GC)	≤ 10%
Heavy metals (ICP-MS)	Iron ≤ 100 ppm;
	Copper ≤ 100 ppm;
	Zinc ≤ 100 ppm;
	Palladium ≤ 100 ppm
Assay (HPLC)	≥50%
Trifluoroacetic acid (GC)	≤40%

HPLC = high performance liquid chromatography; TAMC = total anaerobic microbial count; TYMC = total yeast/mold count; BET = bacterial endotoxin test; LAL = limulus amebocyte lysate; NMR = nuclear MR; GC = gas chromatography; ICP-MS = inductively coupled plasma mass spectrometry.

#### 3.2.S.5 Reference Standards or Materials

A batch of precursor is annually qualified as in-house precursor standard to support the precursor acceptance test.

#### 3.2.S.6 Container Closure System

Once accepted, the PSMA-11 precursor is dissolved and aliquoted into single-use 1.5-mL tubes.

#### 3.2.S.7 Stability

One PSMA-11 precursor vial (e.g., 1 mg) is portioned into 5-μg aliquots. These precursor aliquots are stored in a freezer and used within their expiration date established by the stability study.

### Drug Product

#### 3.2.P.1 Description and Composition of the Drug Product

**FIGURE 1.**
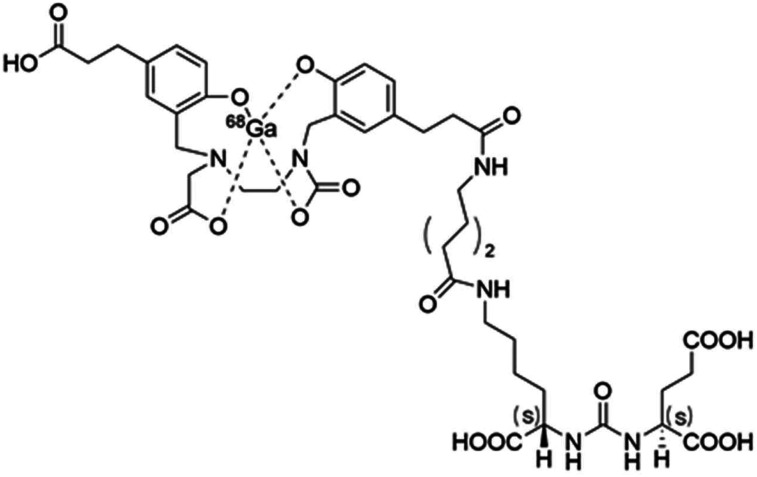
Structural formula of ^68^Ga-PSMA-11.

The structure, chemical name, code, and other nonproprietary names are included in Figure 1 and Supplemental Table 1 (supplemental materials are available at http://jnm.snmjournals.org).

#### 3.2.P.2 Pharmaceutical Development

^68^Ga can be produced by either commercially available generators or by a cyclotron. Each source provides ^68^Ga trichloride in hydrochloric acid.

#### 3.2.P.3 Manufacture

This section lists the manufacturer, the batch formula (component, amount, function, and quality standard), the manufacturing process, control of critical steps and intermediates, and process validation/evaluation (i.e., media fill simulation).

The suitability of the manufacturing process with the E&Z GalliaPharm generator and cyclotron-produced ^68^Ga is demonstrated with 3 consecutive successful quality control releasable runs.

**FIGURE 2.**
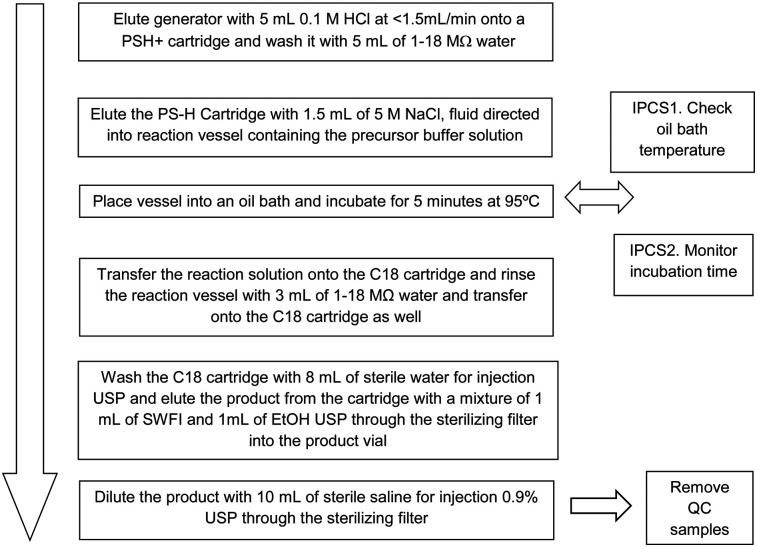
^68^Ga-PSMA-11 production method from UCLA NDA 212642.

For UCLA, 5 μg of freshly thawed precursor in 1.5 mL 1.5 M HEPES (4-(2-hydroxyethyl)-1-piperazineethanesulfonic acid) are mixed with generator-eluted ^68^Ga (5 mL). Then, the vessel containing the reaction mixture is incubated at 95°C for 5 min in an oil bath. At the completion of the reaction phase, the vessel is cooled for 5 min. Then the reaction solution is loaded onto a C18 Sep-Pak Light cartridge (Waters) and washed with sterile United States Pharmacopeia (USP) water and the product eluted with 1 mL of Sterile Water for Injection and 1 mL of Ethanol USP. Finally, the product is diluted with 10 mL of sterile saline for injection 0.9% USP through the sterilizing filter. The process is described in Figure 2. The final formulation is provided in Table 2.

**TABLE 2:** Batch Formula for UCLA Final Product

Component	Amount	Function	Quality standard
^68^Ga-PSMA-11	18.5–185 MBq/mL (0.5–5 mCi/mL)	Drug substance	In-house
PSMA-11	5 μg	Drug substance precursor	GMP
HEPES*	1.5 mL (1.5 M)	Processing aid	PharmaGrade
Hydrochloric acid*^1^	5 mL (0.1 M)	Processing aid	GMP
Sodium chloride 0.9% injection	10 mL	Tonicity	USP
Ethanol	1 mL	Eluent	USP
Water for injection USP	1 mL	Eluent	USP

* HEPES and hydrochloric acid are completely removed during processing and are not present in final formulation. GMP = good manufacturing practice; HEPES = 4-(2-hydroxyethyl)-1-piperazineethanesulfonic acid.

**FIGURE 3.**
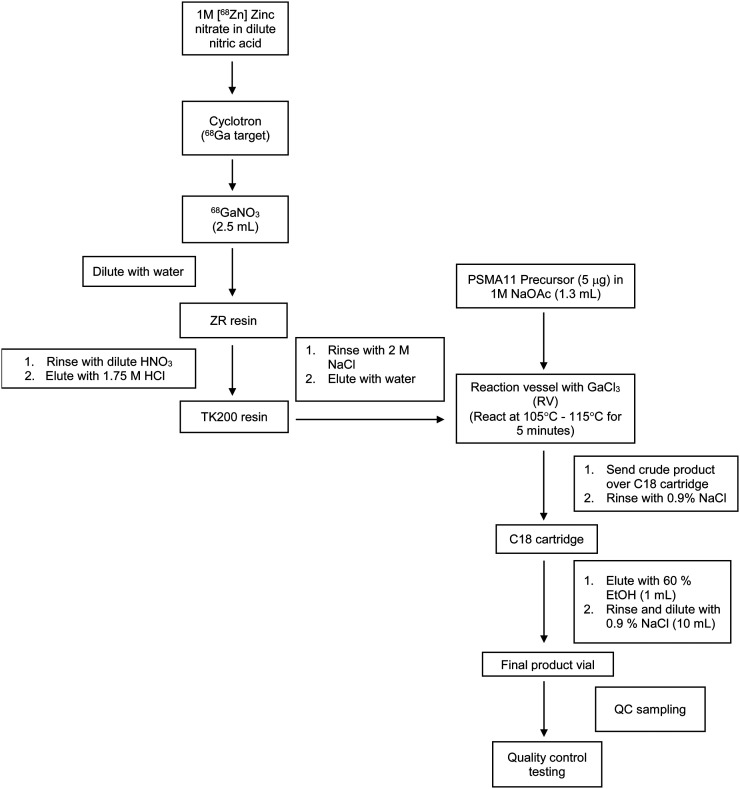
^68^Ga-PSMA-11 production method (cyclotron-based) from UCSF NDA 212643.

For UCSF, ^68^Ga is produced using a PETtrace cyclotron (GE Healthcare). The process involves proton irradiation of a 1 M ^68^Zn-zinc oxide solution in dilute nitric acid. The solution is irradiated for 60 min at a beam current of 35 μA. After irradiation, the ^68^Ga-nitrate solution is delivered to a FastLab (GE Healthcare) synthesis module, where the solution is purified and prepared as ^68^GaCl_3_, which is the same form of ^68^GaCl_3_ eluted from the generator. The ^68^GaCl_3_ is then reacted with 5 μg of PSMA-11 precursor in 0.5 M NaOAc at 100°C–115°C for 5 min. The crude reaction mixture is loaded onto a C18 Sep-Pak Light cartridge, and the cartridge is rinsed with 0.9% sodium chloride for injection USP. The ^68^Ga-PSMA-11 is eluted with 1 mL of 60% ethanol through a 0.22-μm sterilizing filter into the final product vial. Finally, the product is diluted with 10 mL of 0.9% sodium chloride for injection USP through the sterilizing filter. The cyclotron-based process is outlined in Figure 3. The final formulation for cyclotron-based production is provided in Table 3.

**TABLE 3:** Batch Formula for UCSF Final Product (Cyclotron-Based Production)

Component	Amount	Function	Quality standard
PSMA-11	5 μg	Precursor	GMP grade
^68^Ga-PSMA-11	18.5–185 MBq/mL (0.5–5 mCi/mL)	Drug substance	In-house
Sodium acetate, 1 M*	1.3 mL	Buffering agent	Pharma grade
Nitric acid, 0.1 M^1^	4 mL	^68^Ga purification	Trace-metal basis
Hydrochloric acid, 1.75 M*	4 mL	^68^Ga purification	Ultrapure
Sodium chloride, 2 M^1^	4 mL	^68^Ga purification	Trace metal basis
Ethanol, 60%	1 mL	Product eluent	USP grade
Ultra-high purity water	50 mL	Solution preparation	Pharma grade
Water for injection	100 mL	Solution preparation	USP grade
Sodium chloride, 0.9%	5–10 mL	Isotonicity	USP grade

* Completely removed during processing and are not present in final formulation. GMP = good manufacturing practice.

**FIGURE 4.**
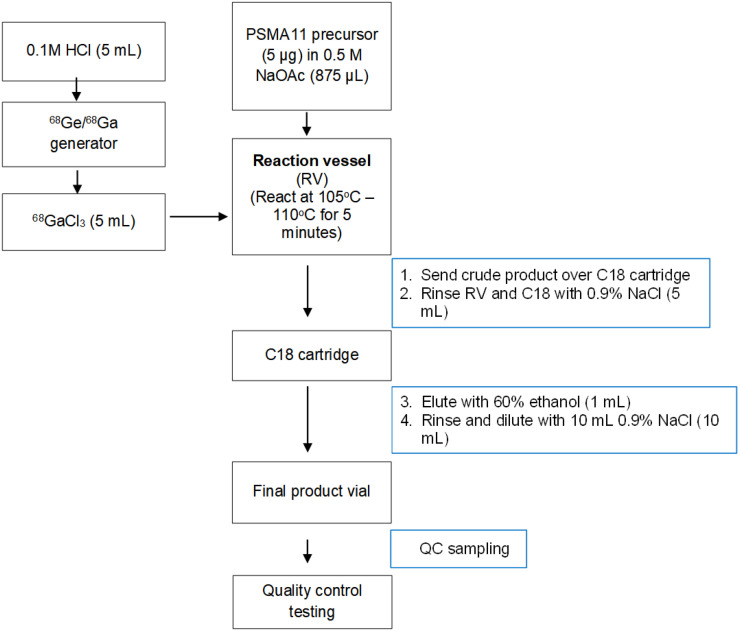
^68^Ga-PSMA-11 production method (generator-based) from UCSF NDA 212643.

For UCSF generator-produced ^68^Ga, 5 μg of freshly thawed precursor in 875 μL 0.5 M sodium acetate trihydrate is mixed with generator-eluted ^68^Ga (5 mL). Then, the vessel containing the reaction mixture is incubated at 105°C for 5 min. At the completion of the reaction phase, the reaction solution is loaded onto a C18 Sep-Pak Light cartridge, washed with 5 mL of 0.9% sodium chloride for injection USP and the product eluted with 1 mL of 60% ethanol USP in water for injection through the sterilizing filter into the product vial. Finally, the product is diluted with 10 mL of 0.9% sodium chloride for injection USP through the sterilizing filter. The generator-based process is described in Figure 4. The final formulation for generator-based production is provided in Table 4.

**TABLE 4:** Batch Formula for UCSF Final Product (Generator-Based Production)

Component	Amount	Function	Quality standard
PSMA-11	5 μg	Precursor	GMP grade
^68^Ga-PSMA-11	18.5–185 MBq/mL (0.5–5 mCi/mL)	Drug substance	In-house
Sodium acetate, 0.5 M*	0.1 mL	Buffering agent	Pharma grade
Hydrochloric acid, 0.1 M*	5 mL	Generator eluent	GMP grade
Ethanol, 60%	1 mL	Product eluent	USP grade
Ultra-high purity water	10 mL	Solution preparation	Pharma grade
Water for injection	4 mL	Solution preparation	USP grade
Sodium chloride, 0.9%	5–10 mL	Isotonicity	USP grade

* Completely removed during processing and are not present in final formulation. GMP = good manufacturing practice.

#### 3.2.P.4 Control of Excipients

Compendial excipients (Supplemental Table 2) are tested and released according to the referenced Pharmacopeia. CoAs for each excipient are usually provided.

#### 3.2.P.5 Control of Drug Product

Every batch of the ^68^Ga-PSMA-11 drug product manufactured for human use conforms to the specified prerelease acceptance criteria as described in Table 5. Brief descriptions of quality control testing methods are provided below.

Appearance

**TABLE 5:** Final Product Specifications

Test	Test method	Acceptance criteria
Prerelease		
Radiochemical purity	Thin-layer chromatography	≥90%
Appearance	Visual observation	Clear solution, free of particulates
pH	pH paper	4.0–7.0
Endotoxin content	PTS Endosafe, USP < 85 >	<17.5 EU/mL
Radiochemical identity	Thin layer chromatography*	UCLA: Rf value = 0.75–0.95
		UCLA: Rf value = 0.3–0.6
Radionuclidic identity	Half-life determination	64.4–71.2 min
Filter integrity	Bubble point pressure reading, USP < 823 > / < 797 >	>50 psi
Postrelease		
Sterility	Direct inoculation. USP < 71 > / < 823 >	Sterile

* UCLA and UCSF use different mobile phases.

The appearance of the ^68^Ga-PSMA-11 is determined by observing the contents of the vial for color, clarity, and particulate matter.

pH

The pH of the final drug product is determined by a colorimetric test. A 1-μL aliquot is spotted on pH indicator paper and then compared with the color reference chart.

Radiochemical Purity

The amount of free ^68^Ga in the radiolabeled ^68^Ga-PSMA-11 preparations is evaluated using instant thin-layer chromatography (iTLC). The stationary phase is an iTLC-silicic acid paper strip. The mobile phase for UCLA is a 50:50 (v/v) solution of 1 M ammonium acetate and methanol and for UCSF 1 M ammonium acetate. Briefly, an approximately 37- to 185-kBq (1–5 μCi) sample of the radiolabeled ^68^Ga-PSMA-11 (used without dilution) is spotted on an iTLC-silicic acid strip and developed in the mobile phase. The test is considered valid if the retardation factor of gallium(III) and gallium in colloidal form is <0.3 and the retardation factor of ^68^Ga-PSMA-11 is 0.75–0.95 (UCLA) or 0.3–0.6 (UCSF).

Radionuclidic Identity

The half-life method is used to determine the identity of the radionuclide. The half-life test result for ^68^Ga should be between 64.4 and 71.2 min.

^68^Ga Radionuclidic Purity

A test is performed annually to determine the ^68^Ga radionuclidic purity and identity using the γ-spectrum obtained with a multiple-channel analyzer. The radionuclidic purity of ^68^Ga is confirmed by absence of peaks on the γ-spectrum that are attributable to radionuclidic impurities and the detection of peaks at 511 keV on the γ-spectrum. Potential contaminants for UCLA drug product are summarized in Supplemental Table 3. Potential contaminants for UCSF drug product are summarized in Supplemental Tables 4 and 5 (generator-based and cyclotron-based, respectively).

Endotoxin Test, USP 85

The ^68^Ga-PSMA-11 final drug product is tested for endotoxin content using the PTS Endosafe system (Charles River Laboratories) according to the manufacturer’s instructions.

Filter Integrity Test, Bubble Point Pressure reading, USP 823/797

The 0.22-μm sterilizing filter is tested to ensure its integrity during filtration of the final drug product. The procedure involves connecting the filter to a gas line with a pressure gauge and submerging into water. The pressure inside the gas line is slowly increased until gas starts to flow through the filter and into the water, producing visible bubbles. The pressure at which the bubbles become visible is recorded. The test is valid if bubbles are visible at or above the filter integrity pressure specified by the filter manufacturer.

Sterility

Sterility testing is performed within 30 h of the drug product’s end-of-synthesis time using the direct inoculation of media method (USP < 823 > for sterility testing of radiopharmaceuticals). Briefly, 1 tube containing Tryptic Soy Broth (TSB) media and 1 tube containing the Fluid Thioglycollate growth media (FTM) are each inoculated with 0.1–0.2 mL of the final drug product. Negative control samples for each type of media are created by inoculating one additional of each type of media tube with 0.1–0.2 mL of sterile water or saline for injection. FTM and TSB samples are incubated for 14 d at 30°C–35°C and 20°C–25°C, respectively, and are observed daily for signs of bacterial growth.

#### 3.2.P.6 Reference Standards or Materials

Supplemental Table 6 shows the references standards used in the control of ^68^Ga-PSMA-11.

#### 3.2.P.7 Container Closure System

Commercial preassembled, sterile, and ready-to-use vials are used. Reference to the DMF for the container/closure system and a representative CoA are provided in the application. ^68^Ga-PSMA-11 is packaged in a presterilized, pyrogen-free primary container closure consisting of a preassembled 20-mL (UCSF) or 30-mL (UCLA) USP Type I glass, a gray butyl preassembled rubber stopper, and an aluminum crimp seal.

#### 3.2.P.8 Stability

An expiration time of 3 h (UCLA) or 4 h (UCSF) after the end of synthesis is used for ^68^Ga-PSMA-11 (based on stability studies). Briefly, after removal of the quality control samples, the final-product vials containing the ^68^Ga-PSMA-11 were positioned at room temperature in an inverted position and stored for 3 h (UCLA) or 4 h (UCSF). After each hour of storage, an additional quality control sample was drawn to reassess appearance, pH, radiochemical identity, and purity by radio-TLC and radio-HPLC (high-performance liquid chromatography). The quality control tests met the acceptance criteria after a 3-h (UCLA) or 4-h (UCSF) storage period of the final product vial in an inverted position.

## CONCLUSION

A joint academic effort has led to the FDA NDA approval for the first drug for PET imaging of PSMA-positive lesions in men with prostate cancer. Although regular drug development and commercialization depends near exclusively on industry investment, academic institutions are indispensable for the regulatory approval of PET drugs. In recent years, FDA granted NDA approvals for Mayo Clinic’s ^11^C-choline, University of Iowa’s ^68^Ga-DOTATOC, and most recently, for Feinstein’s Institute of Medical Research ^18^F-fluorodopa.

UCSF and UCLA teams closely collaborated in their pivotal phase 3 trials, pre-NDA meeting, and NDA filing/amendment. The success of this academic partnership is evidenced by the final NDA approval. The unique parallel regulatory approach paved the way for a collaborative PET drug development by academic institutions. We gained substantial knowledge from our interactions with the FDA’s NDA review team. The FDA’s rigorous but open-minded review process ensured that the efficacy and safety claims were supported by the totality of evidence. Sharing our strategy and experience for and with the FDA regulatory approval process should assist the imaging community to develop strategies for ANDA submissions.

## DISCLOSURE

No potential conflict of interest relevant to this article was reported.
